# Diurnal Variation of Epiphytic Microbiota: an Unignorable Factor Affecting the Anaerobic Fermentation Characteristics of Sorghum-Sudangrass Hybrid Silage

**DOI:** 10.1128/spectrum.03404-22

**Published:** 2022-12-15

**Authors:** Zhihao Dong, Junfeng Li, Siran Wang, Dong Dong, Tao Shao

**Affiliations:** a Institute of Ensiling and Processing of Grass, College of Agro-grassland Science, Nanjing Agricultural University, Nanjing, China; Nanjing Agricultural University

**Keywords:** diurnal variation, epiphytic microbiota, fermentation characteristics, sorghum-sudangrass hybrid

## Abstract

Forage epiphytic microbiota exhibits pronounced changes in composition and function throughout the day. However, the effects of these changes on silage fermentation are rarely explored. Here, we transplanted the epiphytic microbiota of sorghum-sudangrass hybrid (SSG) harvested at 7:00 h (AM), 12:00 h (M), and 17:00 h (PM) to sterilized SSG to evaluate the effects of diurnal variation of epiphytic microbiota on fermentation characteristics. During fermentation, remarkable differences in bacterial community successions were observed between silages inoculated with AM and M microbiota. Compared to AM microbiota, M microbiota inoculation increased the proportions of Pantoea dispersa, Leuconostoc lactis, Enterobacter, and Klebsiella variicola, whereas it decreased the proportions of Weissella cibaria and Lactobacillus plantarum during fermentation. This led to the most rapid pH declines and organic acid production in AM silage and the slowest in M silage. Both M and PM microbiota affected the bacterial cooccurrence patterns, indicated by decreased complexity and stability in the community structures of M and PM silages compared to that of AM silage. The predicted functions indicated that some key carbohydrate metabolism pathways related to lactic acid synthesis were downregulated, while some competing pathways (ascorbate and aldarate metabolism and C5-branched dibasic acid metabolism) were upregulated in M silage compared to AM silage after 3 days of fermentation. Correlation analysis revealed positive correlations between competing pathways and enterobacterial species. The current study highlights the importance of diurnal variation of epiphytic microbiota in affecting the silage bacterial community, potentially providing an effective strategy to improve silage quality by optimizing harvest time.

**IMPORTANCE** Ensiling is a way to preserve wet biomass for animal and bioenergy production worldwide. The fermentation quality of silage is largely dependent on the epiphytic microbiota of the material. Plant epiphytic microbiota exhibit diurnal changes in composition and function. However, the effects of these changes on silage fermentation are rarely explored. The results presented here demonstrated that diurnal variation of epiphytic microbiota could affect the fermentation characteristics and bacterial community during SSG fermentation. Marked bacterial community differences were observed between AM and M silages during the initial 3 days of fermentation. The dominance rate of Lactobacillus plantarum was highest in AM silage, whereas enterobacterial species were more abundant in M silage. The predicted function revealed downregulated lactic acid synthesis pathways and upregulated competing pathways in M silage compared to those in AM silage. This study provides clues for technological-parameter optimization of the fermentation process by the selection of harvest time.

## INTRODUCTION

Silage making is a worldwide practice that uses the natural fermentation carried out by epiphytic microbiota, permitting forage crops to be stored for extended periods. The production of silage is important for agricultural and industrial value chains. It provides not only conserved forage for livestock but also substrates for bioenergy production. In many European countries, silage production accounts for a large share of the feed and substrate supply (1). The biochemistry of silage fermentation is essentially a simple process, but the interaction of microbial and chemical compositions can result in a high degree of variability in fermentation quality. Nevertheless, regardless of the silage production purpose, quality fermentation is essential for the preservation of biomass and its subsequent use.

The quality of fermentation is largely dependent on the epiphytic microbiota naturally present on forage (2, 3). Unlike grain production, the biomass used for silage production is the above-ground part of the plant. It is exposed to the atmosphere and subjected to the diurnal cycle. The populations of many epiphytic microorganisms undergo pronounced changes throughout the day as a consequence of direct impact by environmental fluctuations and indirect influence by plant metabolism (4). It is well established that this turnover rhythm of epiphytic microbiota results in significant changes in composition and function (5). However, possible effects brought by these diurnal changes on silage fermentation are rarely explored, likely because those effects are difficult to differentiate from the effects brought by chemical changes. Recently, silage researchers started to use microbiota transplantation to clarify the role of exogenous epiphytic microbiota in silage quality (2, 6). They demonstrated that exogenous microbiota can reconstruct a similar function in the recipient plant. Moreover, gamma ray radiation is popular in silage research since it can sterilize forage without significantly altering plant chemical compositions and enzyme activities (7). The development of these techniques offers the opportunity to evaluate the effects of exogenous microbiota on silage fermentation.

Growing multipurpose sweet sorghum is gaining popularity, especially in regions that experience drought, delayed planting, and high summer temperatures that limit corn production (8). The sorghum-sudangrass hybrid (SSG; Sorghum bicolor L.× Sorghum sudanense L.) is the most commonly used sorghum type because of its flexible planting time, rapid growth, high yields, and suitability in rotation systems (9). In this study, SSG was selected as the model crop considering its essential role in food, feed, fodder, and fuel security in dryland agriculture. To evaluate the effects of diurnal variation of epiphytic microbiota on SSG silage fermentation, we transplanted epiphytic microbiota of SSG harvested at various times within a day to sterilized SSG. We hypothesized that SSG epiphytic microbiota exhibited variations in composition and function during the daytime and that these changes would affect the fermentation characteristics of SSG. Our study will elucidate the role of diurnal variation of epiphytic microbiota in silage fermentation and advance our ability to produce high-quality silage.

## RESULTS

### Forage characteristics and microbial counts of epiphytic microbiota.

**FIG 1:**
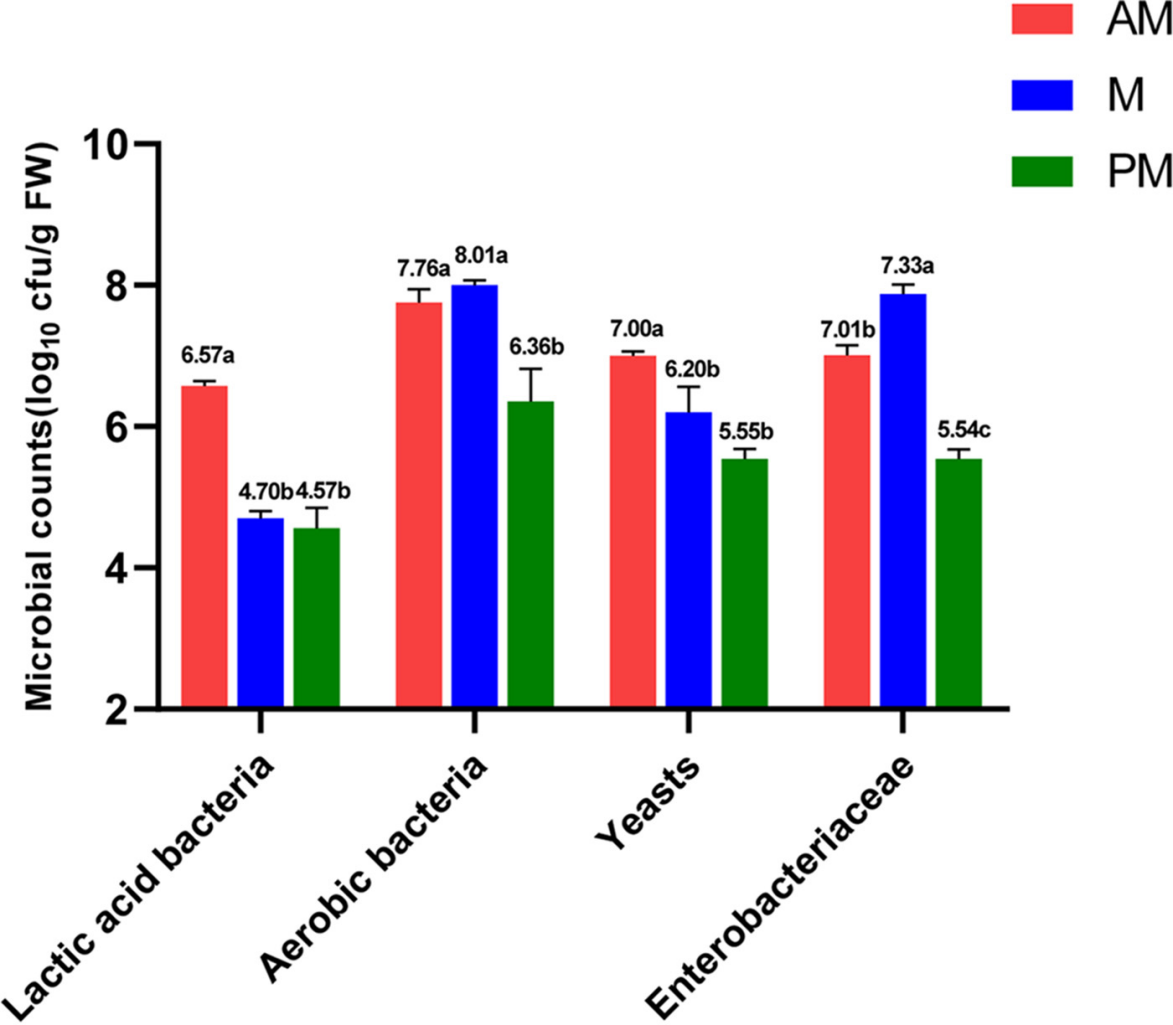
The microbial counts of SSG epiphytic microbiota inoculums prepared from forage harvested at various times of day. CFU counts are shown above the bars. Error bars show standard deviations; different lowercase letters show significant differences. AM, 7:00 h; M, 12:00 h; PM, 17:00 h; FW, fresh weight.

Gamma ray irradiation was used to obtain sterile SSG. As shown by the results in Table 1, for fresh SSG, the dry matter (DM), crude protein (CP), water-soluble carbohydrate (WSC), neutral detergent fiber (NDF), and acid detergent fiber (ADF) contents and buffering capacity (BC) were 208 g/kg fresh weight (FW), 51.3 g/kg DM, 106 g/kg DM, 636 g/kg DM, 411 g/kg DM, and 75.3 meq/kg DM, respectively. The counts of lactic acid bacteria (LAB), aerobic bacteria, yeasts, and *Enterobacteriaceae* of fresh SSG were 2.52, 8.08, 6.70, and 7.60 log_10_ CFU/g FW, respectively. After gamma ray irradiation, no viable microorganisms were detected, suggesting that SSG was entirely sterilized by the gamma ray irradiation. The microbial counts of epiphytic microbiota inoculums prepared from forage harvested at various times of day are presented in Fig. 1. The M and PM microbiota had lower (*P < *0.05) LAB and yeast counts than the AM microbiota. The aerobic bacterial counts were lower (*P < *0.05) in PM microbiota than in AM and M microbiota. The *Enterobacteriaceae* counts were greatest in the M microbiota.

**TABLE 1:** Chemical compositions and microbial populations of fresh and gamma ray-irradiated SSG

Parameter^*a*^	Value for^*b*^:		SEM	*P* value
	FM	IR		
DM (g/kg FW)	208	210	5.56	0.615
CP (g/kg DM)	51.3	53.2	1.48	0.782
WSC (g/kg DM)	106	101	2.29	0.816
NDF (g/kg DM)	636	633	23.7	0.645
ADF (g/kg DM)	411	403	32.4	0.649
BC (mEq/kg DM)	75.3	74.2	12.3	0.747
LAB (log_10_ CFU/g FW)	2.52	ND		
Aerobic bacteria (log_10_ CFU/g FW)	8.08	ND		
Yeasts (log_10_ CFU/g FW)	6.70	ND		
*Enterobacteriaceae* (log_10_ CFU/g FW)	7.60	ND		

a DM, dry matter; FW, fresh weight; CP, crude protein; WSC, water-soluble carbohydrates; meq, milligram equivalent; NDF, neutral detergent fiber; ADF, acid detergent fiber; BC, buffering capacity.

b FM, fresh matter (SSG); IR, gamma ray irradiated SSG; ND, not detected.

### Fermentation characteristics and microbial counts during SSG fermentation.

**FIG 2:**
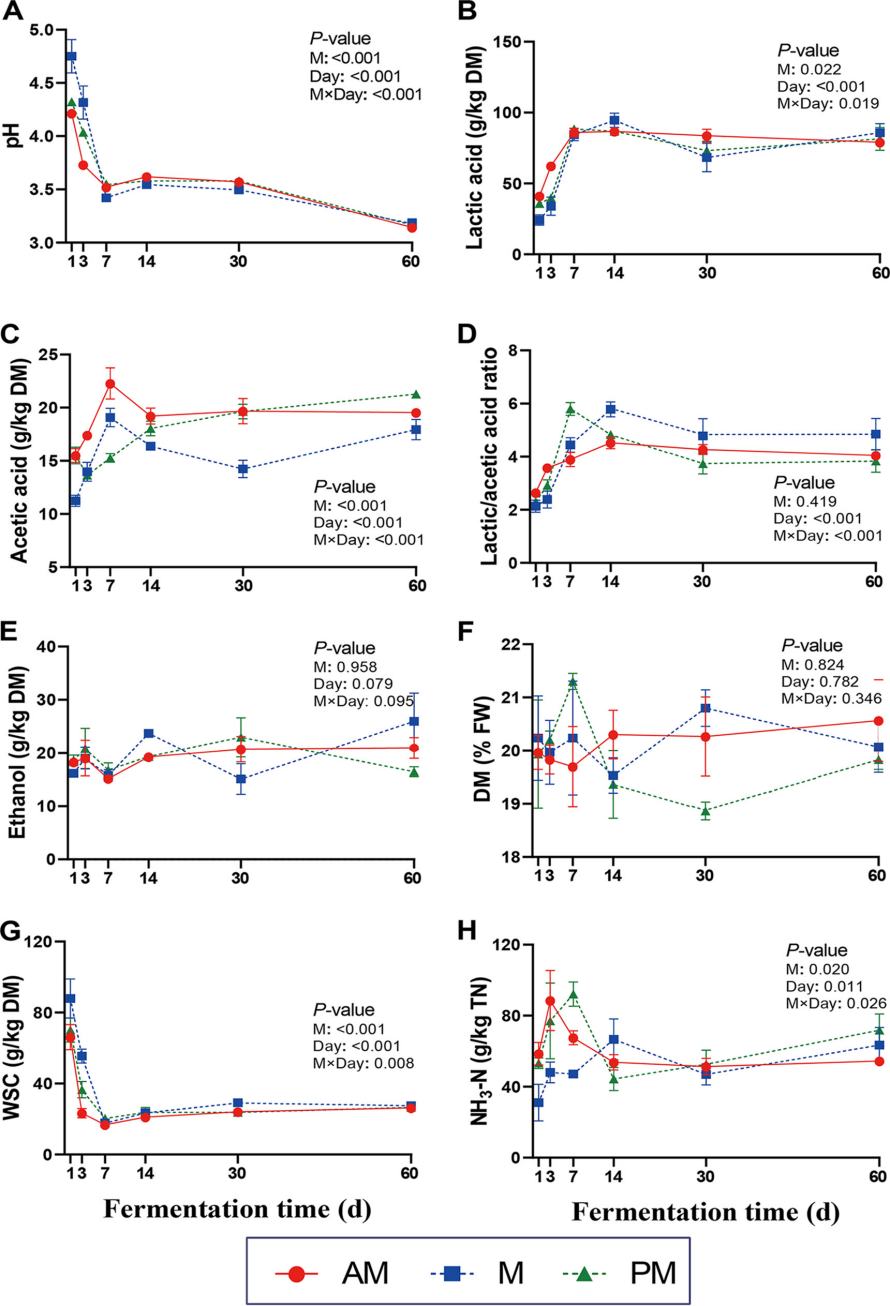
Effects of inoculating epiphytic microbiota from forage harvested at various times of day on fermentation characteristics during SSG fermentation. AM, 7:00 h; M (blue squares), 12:00 h; PM, 17:00 h; DM, dry matter; FW, fresh weight; WSC, water-soluble carbohydrates; NH_3_-N, ammonia nitrogen; TN, total nitrogen; M (inset values), effect of epiphytic microbiota; Day, effect of fermentation time. M×Day, interaction of epiphytic microbiota and fermentation time. Error bars show standard deviations.

The fermentation characteristics of SSG inoculated with epiphytic microbiota from forage harvested at various times of day are given in Fig. 2. The largest declines of pH and greatest increases of lactic acid, acetic acid, and lactic acid-to-acetic acid ratios were observed during the initial 7 days of fermentation. Marked differences in fermentation characteristics were observed among the three silages during this period. As for pH and lactic acid, AM silage exhibited the most rapid pH declines and lactic acid production, followed by PM silage and then M silage. The acetic acid contents were highest in AM silage during the initial 14 days, whereas PM silage had the highest acetic acid contents after 30 days of fermentation. It is worth noting that M silage had the lowest lactic acid-to-acetic acid ratio during the initial 7 days, while it had the highest lactic acid-to-acetic acid ratio after 14 days of fermentation. The propionic acid and butyric acid contents were all detected in trace amounts. The ethanol contents of SSG silages were all lower than 30 g/kg DM, and the DM contents changed little during the fermentation (Fig. 2E and F). There were no differences in ethanol and DM contents among the silages. Consistent with the increases in lactic acid contents, the WSC contents were observed to decline rapidly during the initial 7 days of fermentation. The rates of decline varied among the silages, with the fastest in AM silage, the moderate rate in PM silage, and the slowest in M silage. The NH_3_-N contents peaked between day 3 and day 7 of fermentation and then declined at the late stages of fermentation. All silages contained NH_3_-N at <100 g/kg of total nitrogen (TN), and lower (*P < *0.05) NH_3_-N contents were observed in M silage than in AM silage during the initial 7 days of fermentation.

The microbial counts of SSG inoculated with epiphytic microbiota from forage harvested at various times of day during fermentation are given in Table 2. Marked differences in microbial counts were observed among the three silages during fermentation. In all silages, the LAB counts quickly exceeded 7 log_10_ CFU/g FW after 1 day, peaked between days 3 and 7, and then declined after 7 days of fermentation. The M and PM silages had greater (*P < *0.05) LAB counts than AM silage at day 30, and M silage had greater LAB counts at day 60 of fermentation. As for other microorganisms, there were overall tendencies for them to decrease in the populations during fermentation, but their rates of decrease varied depending on the silage. For AM silage, microorganisms including aerobic bacteria, *Enterobacteriaceae*, and yeasts immediately dropped to below the detection level after the onset of fermentation. In contrast, these microorganisms remained present in large count numbers in the other silages, especially the M silage, for longer periods of time.

**TABLE 2:** Microbial counts of SSG inoculated with epiphytic microbiota from forage harvested at various times of day during fermentation

Type of microbe	SSG microbiota inoculant^*a*^	Microbial count (mean log_10_ CFU/g FW) at indicated fermentation time (days)^*b*^						SEM	*P* value for^*c*^:		
		1	3	7	14	30	60		M	Day	M×day
LAB^*d*^	AM	7.54 a	8.92 a	8.48 a	7.70 a	2.62 bb	2.15 bb	0.012	<0.001	<0.001	<0.001
	M	7.38 a	8.65 a	8.76 a	8.00 a	6.53 ab	6.07 ab				
	PM	7.59 a	8.74 a	8.73 a	8.11 a	6.15 ab	3.38 bb				
Aerobic bacteria	AM	0.00 bb	0.00 bb	1.51 ba	1.65 a	0.82 b	0.98 b	0.342	<0.001	<0.001	<0.001
	M	6.94 aa	7.52 aa	6.32 aa	2.52 b	1.87 b	2.67 b				
	PM	4.40 aa	0.00 bc	0.00 bc	3.41 ab	3.59 a	0.77 bc				
*Enterobacteriaceae*	AM	0.00 b	0.00 b	0.00	1.61	0.00	0.00 b	0.136	<0.001	<0.001	<0.001
	M	6.60 aa	7.12 aa	0.00 b	0.88 b	0.00 b	1.37 ab				
	PM	4.94 aa	0.00 bb	0.00 b	1.70 b	0.00 b	0.00 bb				
Yeasts	AM	0.00 bc	0.00 bc	3.45 ba	2.81 ab	0.70 bab	1.90 bab	0.227	<0.001	0.006	<0.001
	M	6.40 aab	6.94 aa	6.71 aa	3.80 c	5.82 aabc	4.42 abc				
	PM	2.10 b	0.00 b	3.02 b	2.75	1.63 b	2.08 b				

a AM, 7:00 h; M, 12:00 h; PM, 17:00 h.

b FW, fresh weight. Mean values with different lowercase letters (a to b) differ significantly among inoculation groups (*P *< 0.05). Mean values with different small capital letters (A to D) differ significantly among ensiling days (*P *< 0.05).

c M, epiphytic microbiota; Day, fermentation time (days); M×day, interaction between epiphytic microbiota and fermentation time.

d LAB, lactic acid bacteria.

### Bacterial diversity and composition during SSG fermentation.

To reveal the effects of the epiphytic microbiota on the bacterial community and diversity, fermentation was monitored in epiphytic microbiota and silages at days 1, 3, and 60 of fermentation, considering that these time points may better reflect the different stages of fermentation. After quality control, a total of 1,227,751 high-quality reads were obtained in all samples. The average length of the reads was 428 bp. Based on a 97% sequence identity threshold, these reads were clustered into 1,096 operational taxonomic units (OTUs) affiliated with 848 species and 540 genera.

**FIG 3:**
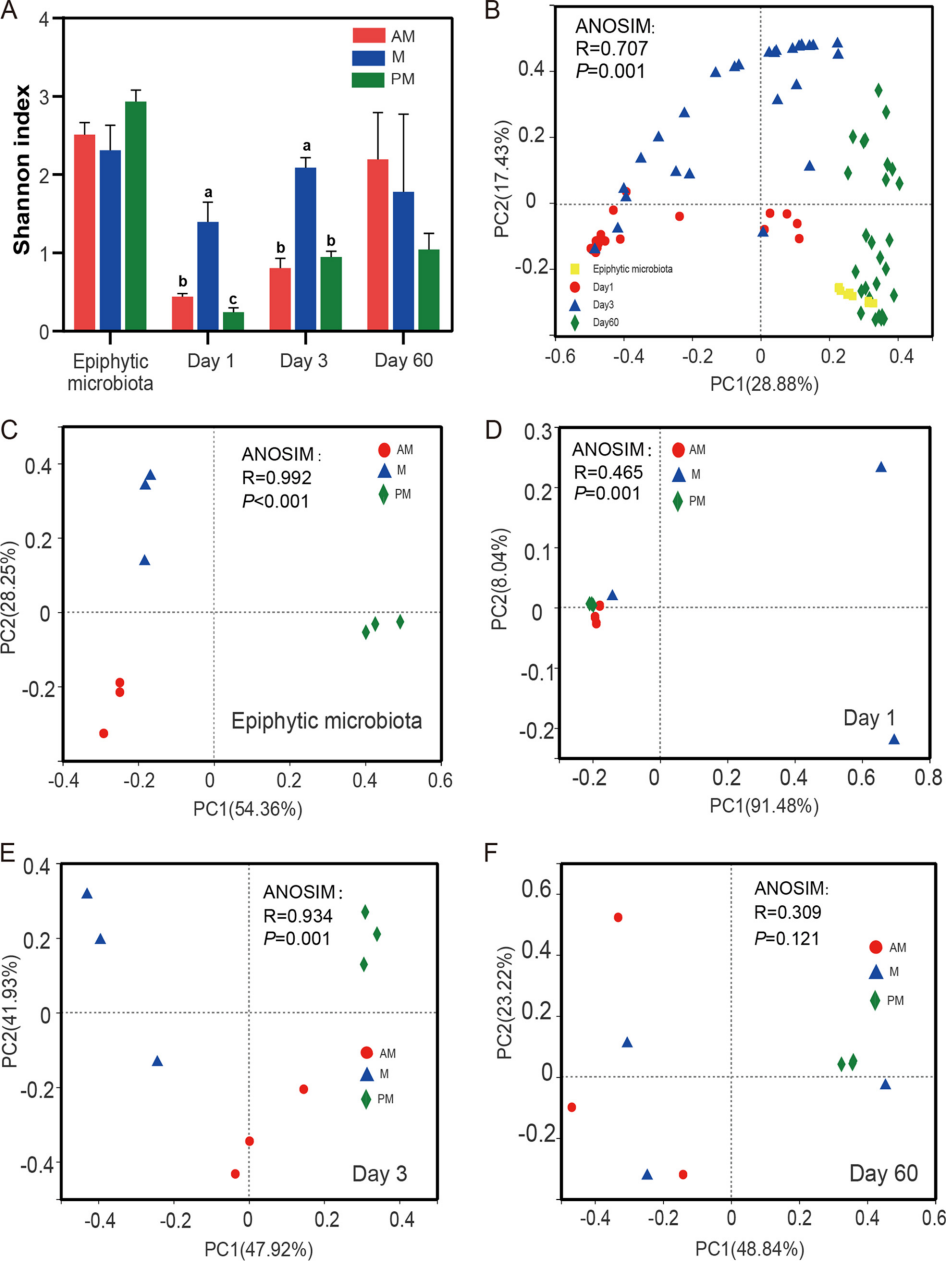
Effects of inoculating epiphytic microbiota from forage harvested at various times of day on bacterial diversity during SSG fermentation. (A) Variations in community α-diversity (Shannon index). Error bars show standard deviations; different lowercase letters show significant differences. AM, 7:00 h; M, 12:00 h; PM, 17:00 h; Day 1, 1 day of fermentation; Day 3, 3 days of fermentation; Day 60, 60 days of fermentation. (B) PCoA of community dissimilarities among fermentation times. (C) PCoA of community dissimilarities of epiphytic microbiota from various harvest times. (D to F) PCoA of community dissimilarities of silages after 1, 3, and 60 days of fermentation.

Bacterial diversity was quantified by means of two metrics, α-diversity and β-diversity (Fig. 3). The M silage had higher (*P < *0.05) Shannon index values at days 1 and 3 of fermentation, suggesting greater α-diversity in M silage than in other silages at the early stages of fermentation (Fig. 3A). To characterize the divergent communities, β-diversity was determined by principal-coordinate analysis (PCoA) (Fig. 3B to F). The PCoA showed that epiphytic microbiota from various times were clearly separated from each other by two axes (Fig. 3C) (analysis of similarity [ANOSIM], *R* = 0.992, *P < *0.001), suggesting a high degree of dissimilarity of epiphytic microbiota compositions. Anaerobic fermentation significantly altered the bacterial communities (Fig. 3B) (ANOSIM, *R* = 0.707, *P = *0.001). According to the β-diversity at each fermentation time, epiphytic microbiota significantly affected the bacterial community compositions during the initial 3 days of fermentation (ANOSIM, day 1, *R* = 0.465, *P = *0.001, and day 3, *R* = 0.934, *P = *0.001) (Fig. 3D and E).

**FIG 4:**
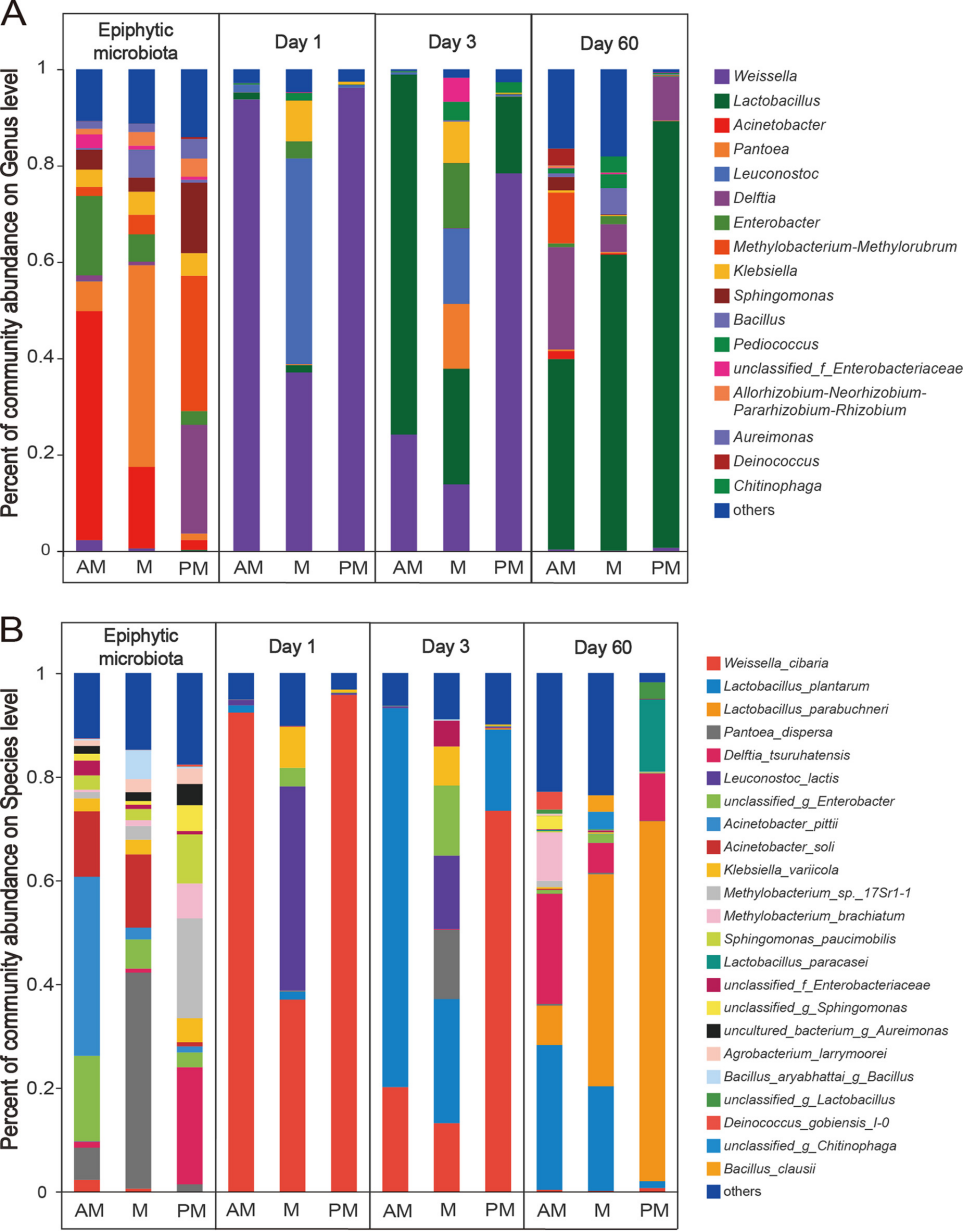
Bacterial community compositions in SSG silages inoculated with epiphytic microbiota from forage harvested at various times of day. (A) Bacterial community composition on the genus level. AM, 7:00 h; M, 12:00 h; PM, 17:00 h; Day 1, 1 day of fermentation; Day 3, 3 days of fermentation; Day 60, 60 days of fermentation. (B) Bacterial community composition on the species level.

The bacterial community compositions at the genus and species levels during SSG fermentation are displayed in Fig. 4A and B. The epiphytic microbiota compositions varied greatly depending on the harvest time. In AM microbiota, the most abundant genera were Acinetobacter (47.5%) and Enterobacter (16.5%). However, the most abundant genera in M microbiota were Pantoea (41.9%) and in PM microbiota were Methylobacterium*-*Methylorubrum (27.8%), Delftia (22.2%), and Sphingomonas (14.5%). At the beginning of fermentation (day 1), Weissella, Leuconostoc, Klebsiella, and Enterobacter were the most abundant genera. As fermentation progressed, the relative abundance of Lactobacillus increased, and the total relative abundance of Weissella and Leuconostoc decreased after 3 days of fermentation. Among the silages, M silage had higher relative abundances of Leuconostoc, Enterobacter, and Klebsiella than other silages during the initial 3 days of fermentation. The relative abundance of Lactobacillus was highest in AM silage after 3 days of fermentation and highest in PM silage after 60 days of fermentation.

On the species level, Acinetobacter pittii (33.9%) and *unclassified_g_*Enterobacter (16.7%) were the most abundant species in AM microbiota, Pantoea dispersa (40.9%) was the most abundant species in M microbiota, and Delftia tsuruhatensis (22.6%), Methylobacterium*_*sp.*_17Sr1-1* (19.2%), and Sphingomonas paucimobilis (9.47%) were the most abundant species in PM microbiota. During the initial 3 days of fermentation, Weissella cibaria, Leuconostoc lactis, Klebsiella variicola, Pantoea dispersa, and *unclassified_g_*Enterobacter were the most abundant species. As fermentation progressed, Lactobacillus plantarum became abundant after 3 days and Lactobacillus parabuchneri became abundant after 60 days of fermentation. Among the silages, M silage had higher relative abundances of Leuconostoc lactis, Klebsiella variicola, and *unclassified_g_*Enterobacter than other silages during the initial 3 days of fermentation. The AM silage had higher relative abundance of Lactobacillus plantarum after 3 days of fermentation, while PM silage had higher relative abundance of Lactobacillus parabuchneri than other silages after 60 days of fermentation.

### Diurnal indicator bacteria and bacterial successions during SSG fermentation.

**FIG 5:**
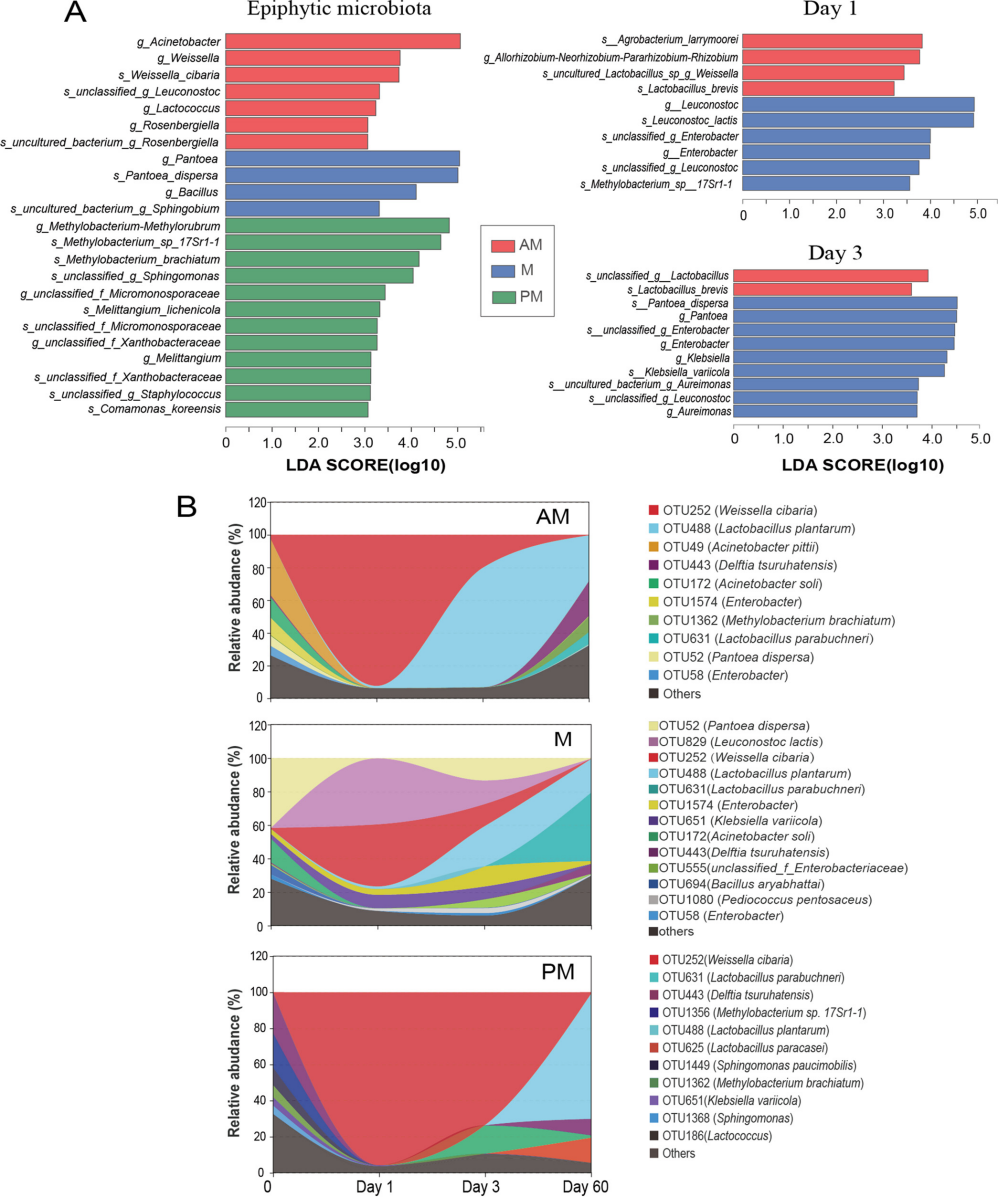
Diurnal indicator bacteria and bacterial successions during SSG fermentation. (A) LEfSe analysis based on bacterial genera and species characterizing bacterial communities among different inoculation groups. AM, 7:00 h; M, 12:00 h; PM, 17:00 h; Day 1, 1 day of fermentation; Day 3, 3 days of fermentation. (B) Stream graphs indicate bacterial community successions on the OTU level in silages inoculated with epiphytic microbiota from forage harvested at various times of day. OTUs with relative abundances of <1% were combined as “Others” in the bacterial succession analyses.

Linear discriminate analysis effect size (LefSe) was used to determine the diurnal indicator bacterial genera and species (Fig. 5A). The indicator bacteria in AM microbiota mainly were Acinetobacter, Weissella, Lactococcus, Rosenbergiella, and Weissella cibaria. Pantoea, Bacillus, and Pantoea dispersa were the indicator bacteria in M microbiota, and bacteria like Methylobacterium*_sp_17Sr1-1*, Methylobacterium-Methylorubrum, and Methylobacterium brachiatum were the indicator bacteria in PM microbiota. During fermentation, diurnal indicator bacteria were detected only in AM and M silages after 1 and 3 days of fermentation. Overall, LAB, such as *s_uncultured_sp_g_*Weissella, *s_unclassified_g_*Lactobacillus, and L. brevis, were the indicator bacteria in AM silage, whereas members of the *Enterobacteriaceae*, including Enterobacter, Klebsiella, Pantoea, *unclassified_g_*Enterobacter, Klebsiella variicola, and Pantoea dispersa, were the indicator bacteria in M silage. In addition, Leuconostoc and Leuconostoc lactis were also specifically enriched in M silage after 1 day of fermentation. The bacterial successions on the OTU level during fermentation are visualized by stream graphs (Fig. 5B). The epiphytic microbiota had remarkable effects on the bacterial successions during SSG fermentation. OTU488 (Lactobacillus plantarum) occupied the AM silage at the highest rate during fermentation. The M microbiota inoculation increased the proportions of OTU52 (Pantoea dispersa), OTU829 (Leuconostoc lactis), OTU1574 (Enterobacter), and OTU651 (Klebsiella variicola) and decreased the proportions of OTU252 (Weissella cibaria) and OTU488 (Lactobacillus plantarum) during fermentation more than the AM microbiota inoculation.

### Bacterial interaction and community stability during SSG fermentation.

**FIG 6:**
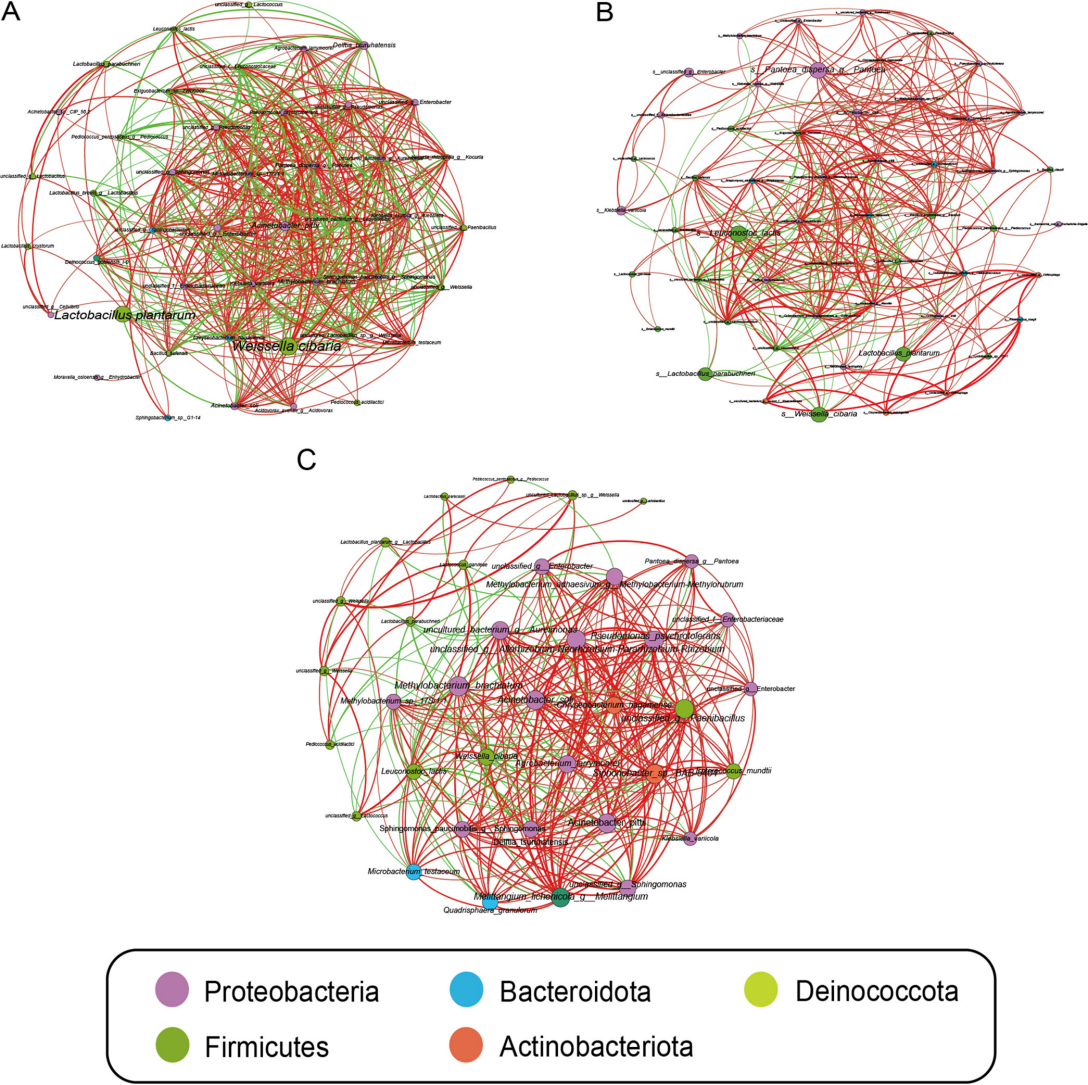
Individual bacterial cooccurrence networks in AM silage (A), M silage (B), and PM silage (C). AM, 7:00 h; M, 12:00 h; PM, 17:00 h. Nodes represent individual OTUs; edges represent significant Spearman correlations (|ρ| > 0.6, *P < *0.05). The size of each node is proportional to the relative abundance, and the nodes are labeled by species and colored by phylum. The thickness of each connection (edge) between two nodes is proportional to the value of the Spearman’s correlation coefficient (|ρ|). The color of the edges corresponds to a positive (red) or negative (blue) relationship.

The individual bacterial network in each silage was examined based on the significant and strong correlations. There were 45, 54, and 37 nodes in the AM, M, and PM networks, respectively, and 25 nodes were shared among them (Fig. 6). Various network indexes, including network size, total edges, average path length, average clustering coefficient, modularity, average degree, graph density, and the ratio of negative/positive interactions, are used to describe the topology properties and structure of networks (Table 3). The total edges and average degrees decreased in M and PM silages compared to those in AM silage, suggesting a decreased complexity of community structures. In addition, lower negative/positive interaction ratios were observed in M and PM silages than in AM silage, indicating a lower stability of bacterial networks during fermentation.

**TABLE 3:** Major topological properties of the molecular ecological networks of bacterial communities in the three silages

Parameter	Value for:		
	AM (7:00 h)	M (12:00 h)	PM (17:00 h)
Network size (*n*)^*a*^	45	54	37
Total no. of edges^*b*^	455	368	301
Avg path length	1.66	2.10	1.74
Avg clustering coefficient	0.74	0.64	0.75
Modularity	0.15	0.33	0.16
Avg degrees	20.2	13.6	16.3
Graph density	0.46	0.26	0.45
Negative edges (%)	31.6	19.8	18.6
Negative/positive ratio	0.42	0.25	0.23

a The number of nodes in a network.

b The number of interactions in the network.

### Predicted functions of bacterial communities during SSG fermentation.

**FIG 7:**
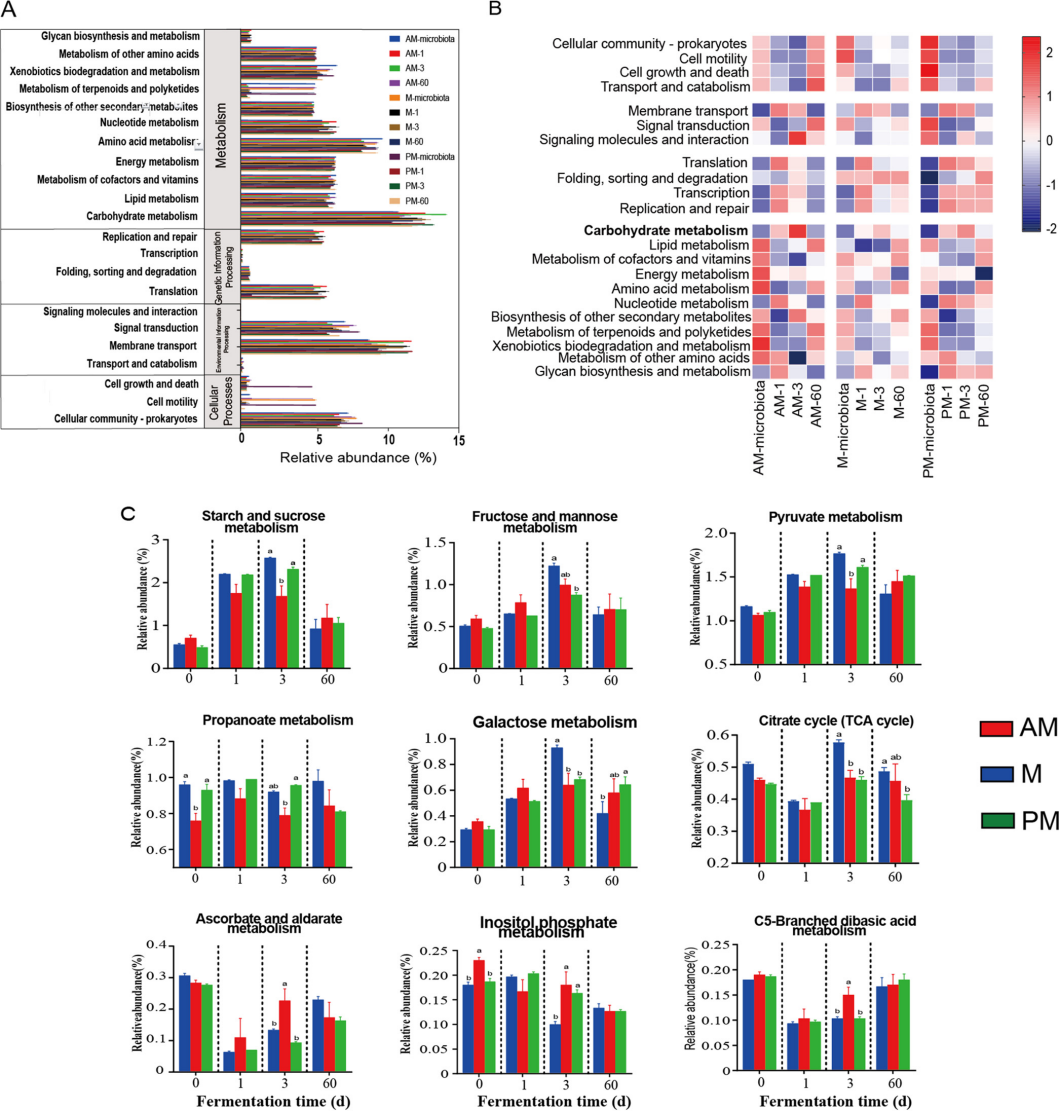
The predicted functions of bacterial communities in SSG silage inoculated with epiphytic microbiota from forage harvested at various times of day. (A) KEGG classification statistics of functional profiles on the second level. Microbiota, epiphytic microbiota; AM, 7:00 h; M, 12:00 h; PM, 17:00 h; 1, 3, and 60, days of fermentation. (B) Comparing taxon-level contribution profiles of functional shifts during fermentation process. (C) Statistical comparison of carbohydrate metabolism pathways on the third level among different inoculation groups. Error bars show standard deviations; different lowercase letters show significant differences.

Tax4fun2 was used to predict the metagenomics functional composition, based on 16S rRNA marker genes. The predicted sequences mainly belonged to metabolism (69.9% to 76.5%), environmental information processing (9.71% to 13.8%), genetic information processing (2.96% to 6.57%), and cellular processes (3.73% to 8.93%) categories (Fig. 7A). In the metabolism category at KEGG level 2, three subfunctions, namely, carbohydrate metabolism (8.58% to 13.9%), membrane transport (5.74% to 10.7%), and amino acid metabolism (5.76% to 7.85%), showed the highest abundances compared to the abundances of other subfunctions. The functional shifts of bacterial communities during SSG fermentation are shown in Fig. 2B. There were remarkable differences in the functional profiles of epiphytic microbiota from various times. The AM microbiota had more abundant metabolism categories, such as lipid metabolism, energy metabolism, amino acid metabolism, and xenobiotic biodegradation and metabolism. The M and PM microbiota had more abundant cell community and cell motility. In addition, cell growth and death, transport and catabolism, and signal molecules and interaction were more abundant in PM epiphytic microbiota. During fermentation, marked upregulations of carbohydrate metabolism were observed after 1 day of fermentation, and the extents of upregulation were distinct among the silages and followed the order AM > PM > M. To investigate the metabolic pathways leading to the differences in carbohydrate metabolism, pathways for processing carbohydrates were further explored at KEGG level 3 (Fig. 7C). Most of the significant pathway differences were observed at day 3 of fermentation. In total, five and three pathways were significantly downregulated in M and PM silages, respectively, compared to AM silage. The most notably downregulated pathways in M silage were starch and sucrose metabolism and pyruvate metabolism and in PM silage were fructose and mannose metabolism and galactose metabolism. Additionally, three pathways, including ascorbate and aldarate metabolism, inositol phosphate metabolism, and C5-branched dibasic acid metabolism, were significantly upregulated in M silage compared to AM silage.

### Correlation analysis of bacterial communities with fermentation characteristics and carbohydrate metabolism pathways.

**FIG 8:**
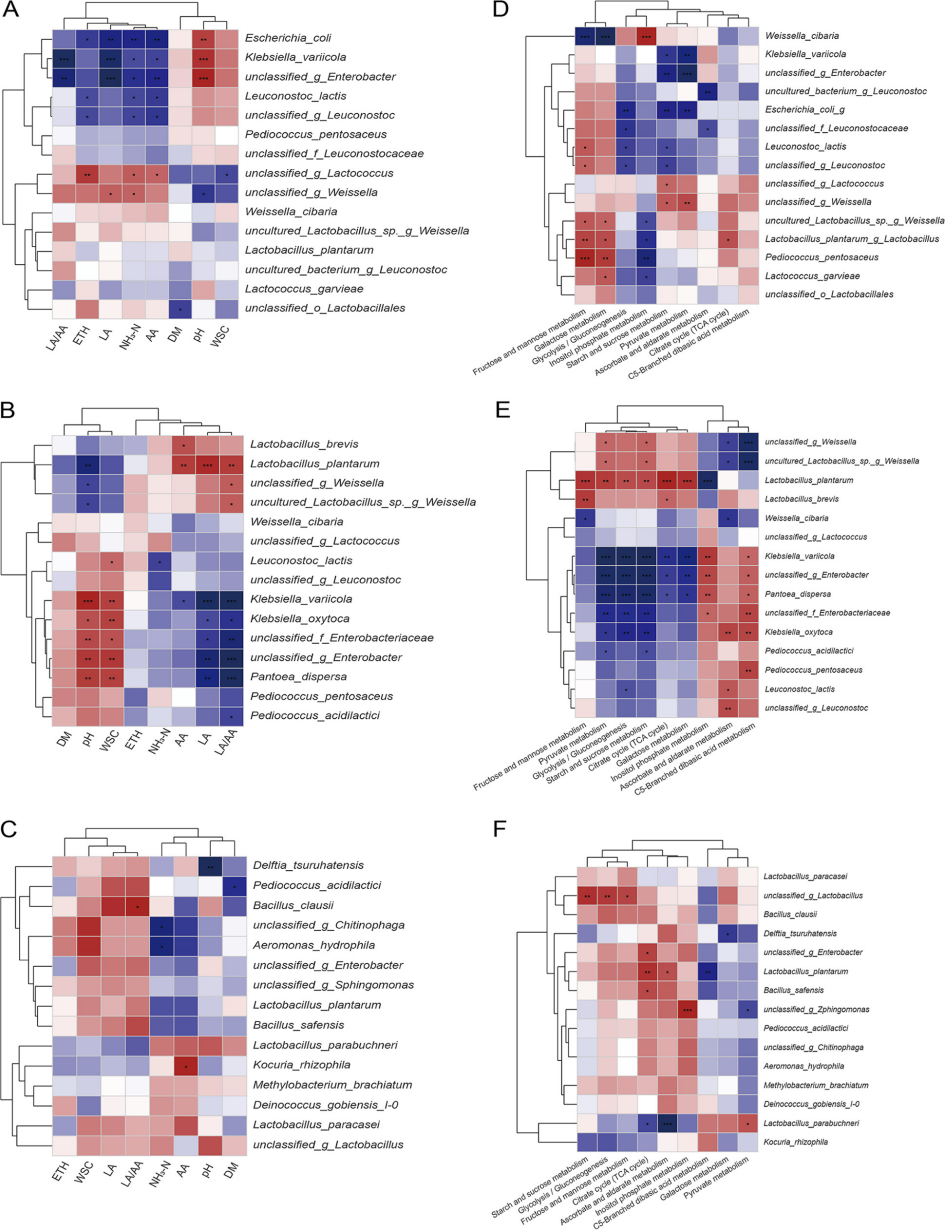
Correlation analysis of bacterial communities with fermentation characteristics (A to C) and carbohydrate metabolism pathways (D to F). The colors in the heat map represent the Spearman correlation coefficient *r*, which ranges between −1 and 1, as shown in the key; *r* < 0 indicates a negative correlation (blue), and *r* > 0 indicates a positive correlation (red). *, *P < *0.05; **, *P < *0.01; ***, *P < *0.001. (A to C) Correlations between bacterial communities and fermentation characteristics on day 1 (A), day 3 (B) and day 60 (C). (D to F) Correlations between bacterial communities and carbohydrate metabolism pathways on day 1 (D), day 3 (E) and day 60 (F).

The correlations of the top 15 bacterial species with the fermentation characteristics and carbohydrate metabolism pathways were explored for each fermentation time (Fig. 8). The correlations between bacterial species and fermentation characteristics and carbohydrate metabolism pathways were strong during the initial 3 days and became weak after 60 days of fermentation. After 3 days of fermentation, enterobacterial species were positively correlated with ascorbate and aldarate metabolism, inositol phosphate metabolism, and C5-branched dibasic acid metabolism. In addition, these enterobacterial species were positively correlated with pH and negatively correlated with lactic acid and acetic acid during the initial 3 days of fermentation. *Unclassified_g_*Lactococcus and *unclassified_g_*Weissella had negative correlations with pH and WSC and positive correlations with acetic acid, NH_3_-N, lactic acid, and ethanol contents after 1 day of fermentation. Moreover, these LAB species also showed positive correlations with starch and sucrose metabolism and pyruvate metabolism. Lactobacillus plantarum was positively correlated with acetic acid, lactic acid, the lactic-to-acetic acid ratio, and a wide range of carbohydrate metabolism pathways and negatively correlated with pH after 3 days of fermentation. After 60 days of fermentation, Kocuria rhizophila was positively correlated with acetic acid and *unclassified_g_*Chitinophaga and Aeromonas hydrophila were negatively correlated with NH_3_-N.

## DISCUSSION

The current study explored the influences of diurnal variation of epiphytic microbiota on the fermentation characteristics of SSG through gamma ray irradiation and microbiota transplantation. In the present study, gamma ray irradiation at a sufficient dose entirely sterilized the forage, which provided an ideal condition for studying the effects of exogenous microbiota inoculation on silage fermentation characteristics. The WSC content of the raw material is another important factor in silage fermentation (10). In line with our previous study, the WSC content of SSG in the present study satisfied the requirement (>60 g/kg DM) for quality fermentation (11). It suggested the benefit of using SSG as the plant material in silage production.

The epiphytic microbiota, the microorganisms naturally present on the surface of plants, is responsible for silage fermentation (3). However, the aerial parts of plants form a highly diverse and dynamic environment where epiphytes need to cope with UV radiation exposure, low water and nutrient availability, and high temperature fluctuations throughout the day (12). Epiphytic microbiota compositions exhibit considerable variations in diurnal and temporal patterns (5). The epiphytic microorganisms of greatest concern in fermentation are LAB, enterobacteria, aerobic bacteria, and yeasts. Because silage is preserved by lactic acid fermentation, only epiphytic LAB are beneficial; others are considered undesirable, causing either fermentation failure or silage deterioration. The present results showed that epiphytic LAB counts decreased significantly in M and PM microbiota compared to the counts in AM microbiota. This was not surprising, since LAB lack many of the microbial traits that are found in phyllosphere-adapted bacteria. For example, many phyllosphere bacteria are pigmented and use these pigments to protect against high levels of UV radiation on leaf surfaces (13, 14). Similarly, Li et al. (15) reported that 8 h of UV exposure significantly decreased the LAB counts on Siberian wildrye. The yeast count also decreased during the day, suggesting their high sensitivity to the dynamic diurnal environment. However, this was inconsistent with our previous observation in Napier grass, where the yeast counts did not change significantly (11). The discrepancy could be associated with the differences in leaf physical structures and nutrient conditions between the two forage crops (16). The M microbiota had the highest *Enterobacteriaceae* counts, which was consistent with the enrichment of Pantoea. Pantoea is a genus of Gram-negative bacteria belonging to the family *Enterobacteriaceae*. Unlike LAB, Pantoea are yellow pigmented, motile, and grow in mucoid-forming colonies. These traits make them successful inhabitants in the phyllosphere (17). The dominance of Pantoea in M microbiota suggested that the harsh environment during the day may have an effect of enriching stress-resistant species in the phyllosphere.

Silage fermentation depends on epiphytic LAB converting WSC to organic acids, mainly lactic acid, along with reduction of the pH. It is generally accepted that at least 5 log_10_ CFU/g LAB is required for adequate fermentation (10). Therefore, the differences in the epiphytic LAB populations may explain the varying rates of pH declines and organic acid production among the silages during fermentation. However, this could not explain the fermentation differences between M and PM silages, considering the comparable epiphytic LAB counts. One explanation could be the high enterobacterial number in M microbiota compared to the number in PM microbiota. Enterobacteria are principal competitors of LAB for available sugars during fermentation (18). The diurnally enriched enterobacteria may increase their competition with LAB, restrain LAB development, and further retard the fermentation. Likewise, Östling and Lindgren (19) have reported that inoculating a crop with enterobacteria resulted in temporarily retarded rates of lactic acid and acetic acid production. The acetic acid contents were observed to be highest in PM silage after 30 days of fermentation, suggesting a greater activity of acetic acid-producing bacteria, such as heterolactic acid bacteria. The lactic acid-to-acetic acid ratio is an indicator of the extent of homofermentation in relation to heterofermentation during fermentation (20). In the present study, all SSG silages had a ratio of lactic acid to acetic acid of >2.0, suggesting the predominance of homofermentation during SSG fermentation. The lactic acid-to-acetic acid ratio was higher in M silage after 14 days of fermentation, suggesting stronger homofermentation than in the other silages.

Ethanol has little preservation effect and its production is associated with extremely high DM and energy losses in silage. More than 30 to 40 g/kg DM of ethanol production in silage is mainly associated with the action of yeasts (21). In the present study, all SSG silages had ethanol contents of <30 g/kg DM, suggesting that ethanol was mainly produced by microbes like heterolactic acid bacteria and enterobacteria. The NH_3_-N content is an indicator of the extent of protein breakdown in silage (22). The NH_3_-N contents of all silages were within the acceptable ranges of NH_3_-N (<100 g/kg of TN), suggesting that SSG protein was well preserved during fermentation. Generally, NH_3_-N would accumulate continuously during fermentation. However, our results indicated that NH_3_-N contents decreased at the late stages of fermentation. A similar phenomenon has been reported by Ogunade et al. (23). In that study, they ascribed it to the oxidization of NH_3_-N under low dissolved oxygen conditions by some ammonia-oxidizing bacteria. Acidification by organic acids produced during fermentation is the main means of controlling the growth of undesirable microorganisms in silage. Therefore, it is not surprising that the populations of undesirable microorganisms, including aerobic bacteria, *Enterobacteriaceae*, and yeasts, showed overall tendencies to decrease during fermentation. The undesirable microorganisms remained in large counts in M silage for longer periods of time, which could be due to its slowest pH decline and organic acid production during fermentation.

Generally, diverse bacterial communities in crops are formed in the field and LAB development will simplify the bacterial community and result in a decline in alpha diversity during fermentation (24). Alpha diversity was low during the early stages of SSG fermentation and increased at the late stages of fermentation. This could be closely related to the changes in the LAB population; LAB multiplied extensively during the early stages of fermentation, reached the maximum number through 3 to 7 days of fermentation, and then kept decreasing in the later fermentation period. The M silage had higher alpha diversity than the other silages during the initial 3 days of fermentation. This was possibly due to its having the slowest pH decline, which did not effectively inhibit the undesirable microorganisms (25). According to the PCoA results, the bacterial community compositions at days 1 and 3 of fermentation were clearly separated by different inoculation groups. This suggested that the diurnal variation of epiphytic microbiota significantly affected the bacterial community successions during the early stages of SSG fermentation.

The competition between LAB and undesirable microorganisms takes place during fermentation, and an anaerobic fermentation dominated by LAB is the key to producing a well-preserved silage (26). Weissella and Leuconostoc were the first dominant LAB genera during SSG fermentation. These LAB species are the most isolated from standing plants, carrying out mixed acid fermentation and contributing to the initial pH decline during fermentation (27). Weissella was identified as the indicator LAB in AM silage, whereas Leuconostoc was the indicator LAB in M silage at day 1 of fermentation. It suggested that the varying rates of acidification may result in the establishment of different niches that are favorable for different LAB species (28). After 3 days of fermentation, more-acid-tolerant Lactobacillus began to dominate the fermentation, along with the decreases in the relative abundances of Weissella and Leuconostoc. The Lactobacillus bacteria mainly consisted of Lactobacillus plantarum, which is the most frequent LAB species in anaerobic plant matter, as well as in many fermented food products (29). Lactobacillus plantarum generally contributes to the substantial lactic acid accumulation and desirable fermentation properties in silage because of its acid-resistant nature and superior ability to utilize a wide variety of substrates. The fastest dominance of Lactobacillus plantarum was consistent with the most rapid and intense lactic acid fermentation in AM silage. The relative abundance of Lactobacillus parabuchneri was greater in PM silage after 60 days of fermentation. Lactobacillus parabuchneri is a heterofermentative LAB species producing acetic acid as its main product, and in addition, it can convert lactic acid into acetic acid and 1,2-propanediol in silage (30). These facts may partly explain the highest acetic acid contents in PM silage after 30 days of fermentation.

Enterobacteria were the primary undesirable bacteria that showed differences in abundance in epiphytic microbiota and silages among different inoculation groups. The dominant enterobacteria in the epiphytic microbiota were mainly Pantoea species, whereas they were rapidly replaced by Enterobacter, Klebsiella, and Escherichia coli after the beginning of fermentation. This was consistent with the report of Li and Nishino (31) that the enterobacteria found on fresh grass would be replaced by enterobacterial species, which adapt better to the silage environment. After 3 days of fermentation, Pantoea was again detected in large abundance, suggesting that some members of the Pantoea genus could survive the anaerobic, acid environment. Similarly, Liang et al. (32) reported that Pantoea bacteria were detected apparently at early and late stages of *paocai* fermentation. Among the silages, enterobacteria, including Enterobacter, Klebsiella, and Pantoea, were specifically enriched in M silage at the early stages of fermentation. It is generally accepted that a rapid pH decline is the key to controlling enterobacteria in silage (33). Their prosperity could be due to the retarded fermentation that allowed them to persist longer in the silage. Enterobacter is a common genus during natural fermentation of various forage crops (34). A higher relative abundance of Enterobacter could enhance the ammonia and biogenic amine production by deaminating and decarboxylating amino acids in silages (35). Klebsiella species can destabilize silage aerobic stability, and some species are opportunistic pathogens that can cause mastitis in animals (36). The role of Pantoea species in silage fermentation is less known. However, researchers consider that they have a role similar to that of Enterobacter species, competing with LAB for available sugars during fermentation (37).

Microbial communities are shaped by interactions among the populations that affect the community dynamics and function (38). The exploration of cooccurrence networks offered new insight into the structure of complex microbial communities. The results demonstrated that the complexity of the bacterial networks decreased in M and PM silages compared to the bacterial network in AM silage. Hernandez et al. (39) pointed out that the complexity of microbial networks would decrease along with the stress gradient. These results suggested that the stressful environment during the day may have acted as a strong filtering factor against the existing epiphytic bacterial species and affected the species cooccurrence patterns during fermentation. Butler and O’Dwyer (40) suggested that positive pairwise interactions can push communities closer to instability. The stressed bacterial communities had lower ratios of negative/positive interactions during fermentation, suggesting the lower stability of communities. The lower ratios of negative/positive interactions in the cooccurrence networks could be due to some “competitive” taxa that engaged in antagonistic interspecific interactions during fermentation being replaced by stress-tolerant species (e.g., oligotrophic microbes). For example, some OTUs affiliated with LAB species, such as *unclassified_g_*Lactococcus and *unclassified_g_*Weissella, were replaced by bacterial species like Pantoea dispersa or members of Methylobacterium*-*Methylorubrum (Fig. 6).

In accordance with our previous study, carbohydrate metabolism, membrane transport, and amino acid metabolism are the main metabolic pathways involved in silage fermentation (41). The epiphytic microbiota from various times had remarkably different functional profiles, suggesting their different metabolic potentials. The M and PM microbiota had more abundant cell community and greater cell motility than the AM microbiota. The increased abundances of these pathways could be associated with the enrichment of stress-resistant bacteria in SSG during the day. Different epiphytic microbiota induced various extents of upregulation of carbohydrate metabolism during fermentation, reflecting their different capacities in metabolizing WSC. Some carbohydrate metabolism pathways were significantly downregulated in M and PM silages compared to their regulation in AM silage after 3 days of fermentation. These results suggested that the loss of function of the bacterial communities in metabolizing some types of carbohydrates contributed greatly to delayed fermentation in the silages. Notably, among the downregulated pathways, starch and sucrose metabolism and pyruvate metabolism were especially downregulated in M silage. Starch and sucrose are the major storage forms of carbohydrate in sorghum (42), and pyruvate is the precursor for the generation of organic acids like lactic acid, α-acetolactate, acetic acid, and formic acid (43). The downregulation of metabolism pathways related to those key carbohydrates and metabolites may strongly limit lactic acid fermentation, possibly explaining why fermentation was retarded to the greatest extent in M silage. Apart from the downregulated pathways, some carbohydrate metabolism pathways were observed to be upregulated in M silage compared to AM silage. These included ascorbate and aldarate metabolism, inositol phosphate metabolism, and C5-branched dibasic acid metabolism. Yin et al. (44) reported that metabolism of ascorbate and aldarate and C5-branched dibasic acid would consume large amounts of sugars. They suggested that the presence of these pathways would compete for the available sugars and shift the carbon flux away from lactic acid synthesis.

The flourishing of enterobacteria would deplete carbohydrate reserves and result in the failure of LAB to dominate fermentation (45). After 3 days of fermentation, enterobacterial species were positively correlated with ascorbate and aldarate metabolism, inositol phosphate metabolism, and C5-branched dibasic acid metabolism. This confirmed that they were the main substrate competitors of LAB during the fermentation. Among the enterobacterial species, Pantoea dispersa was also positively correlated with the competing pathways, suggesting that diurnally enriched Pantoea bacteria could increase the competitive pressure on LAB during fermentation. Enterobacterial species are sensitive to pH declines (22). Therefore, they were positively correlated with pH and negatively correlated with lactic acid. Besides lactic acid, acetic acid also showed negative correlations with the enterobacterial species at day 1 of fermentation. Acetic acid is known to retard enterobacterial growth in silage (45). Negative correlations between acetic acid and enterobacterial species suggested its important role in inhibiting enterobacterial growth at the initial stage of fermentation.

After 1 day of fermentation, acetic acid and lactic acid were positively correlated with *unclassified_g_*Lactococcus and *unclassified_g_*Weissella. Moreover, these LAB species also showed positive correlations with starch and sucrose metabolism and pyruvate metabolism. These results suggested that they played key roles in the production of organic acids and the control of enterobacteria at the initial stages of fermentation. Lactobacillus plantarum contains a comprehensive carbohydrate utilization system composed of plentiful sugar uptake- and metabolism-related genes that endow it with its strong carbohydrate utilization ability (46). After 3 days of fermentation, Lactobacillus plantarum were positively correlated with acetic acid, lactic acid, the lactic acid-to-acetic acid ratio, and a wide range of carbohydrate metabolism pathways. This confirmed the key role of Lactobacillus plantarum in extensive carbohydrate metabolism and the production of organic acids during the fermentation process. After 60 days of fermentation, the few correlations between bacterial species and fermentation characteristics indicate the decreasing effect of bacterial community on fermentation at the late stages. However, it was observed that Kocuria rhizophila was positively correlated with acetic acid after 60 days of fermentation. Kocuria rhizophila possesses a complete set of genes for acetate catabolism via the glyoxylate cycle pathway (47). The positive correlation suggested that high acetic acid content may favor the survival and growth of Kocuria rhizophila in SSG silage. Aeromonads preferentially use ammonium as their nitrogen source (48), and Chitinophaga bacteria were identified as the nitrifiers in activated sludge under low dissolved oxygen conditions (49). Negative correlations of Aeromonas hydrophila and *unclassified_g_*Chitinophaga with NH_3_-N suggested that these bacteria may be responsible for the decreased NH_3_-N contents at the late stages of fermentation.

### Conclusion.

Gamma ray irradiation and microbiota transplantation offered the opportunity to evaluate the effects of diurnal variation of epiphytic microbiota on silage fermentation. The results showed that the epiphytic microbiota of SSG harvested at various times of the day varied greatly in composition and function. The diurnal variation affected the competition between LAB and enterobacteria, leading to increased proportions of Pantoea dispersa, Leuconostoc lactis, Enterobacter, and Klebsiella variicola, whereas the proportions of Weissella cibaria and Lactobacillus plantarum in M silage decreased during fermentation compared to their proportions in AM silage. Marked differences in fermentation characteristics were observed among the silages during the initial 7 days of fermentation, with the fastest pH declines and organic acid production in AM silage and the slowest in M silage. Both M and PM silages exhibited decreases in the complexity and stability of bacterial networks compared to those in AM silage during the fermentation. Our study reveals the importance of diurnal variation of epiphytic microbiota in silage fermentation, which provides clues for technological parameter optimization for the fermentation process.

## MATERIALS AND METHODS

### Experimental material and inoculum preparation.

The sorghum-sudangrass hybrid (SSG; Sumu no. 03) was grown at Nanjing Agricultural University (32°01′19″N, 118°51′08″E, 25 m above sea level), Nanjing, China. The SSG was planted on 23 April 2021 in nine experimental plots (8 m by 5 m each), and all plots had the same tillage, irrigation, and fertilization practices. After 12 weeks of growth, these plots of SSG were randomly assigned to three harvest times (7.00 h [AM], 12.00 h [M], and 17.00 h [PM]). Thus, this study had three sources of forage: AM-, M-, and PM-harvested SSG. At each harvest time, the temperature and relative humidity were measured with a temperature and humidity recorder (UT331; Youlide Instruments Co., Ltd., Zhengzhou, China), and the solar radiation intensity was recorded with a portable pyranometer (Metravi 207; Metravi Instruments Pvt. Ltd, West Bengal, India). The temperatures at the AM, M, and PM times were 26.9, 29.2, and 25.5°C, the relative humidity levels were 93.8, 86.1, and 95.2%, and the solar radiation intensities were 95.2, 312, and 152 W/m^2^, respectively.

The harvested SSG was chopped into lengths of ~1 to 2 cm for inoculum preparations. The inoculum of microbiota was prepared according to the method of Mogodiniyai Kasmaei et al. (2), with modifications. Briefly, a volume of 1,200 mL of Ringer solution fortified with Tween 80 at 0.5 mL/L was mixed with 333 g of SSG. The mixture was then kept in the orbital shaker at 120 rpm for 60 min and filtered with four layers of cheesecloth. The filtrate was centrifuged at 15,500 × *g* for 10 min. The supernatants were discarded, and the pellet was resuspended in 3 mL quarter-strength Ringer solution. Additionally, another batch of SSG was harvested, chopped (~1 to 2 cm), and used for silage preparation. Amounts of about 300 g of SSG were vacuum packaged into polyethylene plastic bags (30 by 40 cm). In total, 54 samples (3 inoculums × 6 storage periods × 3 replicates) were prepared and irradiated with gamma radiation at 32 kGy over 2 h using a ^60^Co source (Nanjing Xiyue Irradiation Technology Co., Ltd, Nanjing, China). The irradiated bags were opened in a laminar flow cabinet and inoculated with different sources of epiphytic microbiota (i.e., AM, M, and PM microbiota). According to the method of Mogodiniyai Kasmaei et al. (2), there were two assumptions for the inoculum: (i) the microbial population was removed entirely from the fresh forage and was evenly distributed in the liquid fraction, and (ii) the recovery of the microbial population from centrifugation was 90%. Therefore, the eluted inoculum (3 mL) represented the whole epiphytic bacterial population from 300 g of fresh forage. After inoculation, the bags were resealed and stored at room temperature (20 to 25°C). The bags were opened after 1, 3, 7, 14, 30, and 60 days of fermentation for chemical and microbial analyses.

### Experimental analyses.

The pre-ensiling forage and silages were thoroughly mixed before chemical analyses. Approximately 100-g samples were oven dried for 48 h at 60°C for dry matter (DM) determination. After that, dried samples were ground with a laboratory pulverizer (FW100; Taisite Instrument Co., Ltd., Tianjin, China) to pass through a 1-mm screen for total nitrogen (TN), water-soluble carbohydrate (WSC), neutral detergent fiber (NDF), and acid detergent fiber (ADF) measurements according to the methods of Dong et al. (10). The crude protein (CP) content was calculated by multiplying TN by 6.25. The DM contents were corrected with the losses of volatiles during oven drying using the equations of Gallo et al. (50).

To determine the ensiling traits of fresh material and fermentation parameters of silage, ~20-g samples were blended with 180 mL distilled water and macerated for 24 h at 4°C. The extracts were filtered through 2 layers of cheesecloth and a filter paper. The pH was measured with a Hanna HI 2221 pH meter (Hanna Instruments Italia Srl, Villafranca Padovana, Italy). The buffering capacity of fresh material and ammonia nitrogen (NH_3_-N) content of silage were determined using the methods of Dong et al. (10). The organic acids (including lactic, acetic, propionic, and butyric acids) and ethanol were quantified using an Agilent 1260 high-performance liquid chromatography (HPLC) system equipped with a refractive index detector (Carbomix H-NP5 column, 2.5 mM H_2_SO_4_, 0.5 mL/min).

For microbial population analyses, 10-g samples were thoroughly blended with 90 mL of sterilized saline solution on a shaker at 120 rpm for 90 min. One hundred microliters of the blended liquid was serially diluted with sterilized saline solution. The LAB, aerobic bacteria, *Enterobacteriaceae*, and yeast counts were determined according to the methods of Dong et al. (10). After that, the remaining blended liquid was filtered into a 50-mL centrifuge tube with 4 layers of cheesecloth. The blended liquid was centrifuged at 4°C for 15 min at 10,000 rpm. The supernatant was discarded, and the pellet was used for DNA extraction.

### Bacterial diversity and community analysis.

The DNA extraction was conducted using the FastDNA spin kit and the FastPrep instrument (MP Biomedicals, Santa Ana, CA) according to the manufacturer’s protocols. The quantity and quality of the DNA obtained were determined using the NanoDrop 2000 UV-Vis spectrophotometer (Thermo Scientific, Wilmington, USA). Universal primers 338F and 806R were used for the PCR amplification, with the target being the V3-V4 region of the bacterial 16S rRNA gene. The PCR products were purified using the AxyPrep DNA gel extraction kit (Axygen Biosciences, Union City, CA, USA) and quantified using QuantiFluor-ST (Promega, USA) according to the manufacturer’s protocol. The DNA were paired-end sequenced (2 × 300 bp) on an Illumina MiSeq PE300 platform (Illumina, Inc., San Diego, CA) at Majorbio Bio-Pharm Technology Co., Ltd., Shanghai, China.

Raw sequences were processed using FLASH (version 1.2.11). The QIIME (version 1.9.1) quality control process was used to discard low-quality sequences (quality scores of <20). Chimeric sequences were identified and removed using UCHIME (version 1.7.0). Only sequences at least 200 bp long after quality filtering were grouped into operational taxonomic units (OTUs) at the 97% similarity level. The α-diversity estimators (Shannon, abundance-based coverage estimator [ACE], Chao1, and coverage indexes) were analyzed using QIIME (version 1.9.1). The β-diversity analysis, performed by principal coordinate analysis (PCoA), was used to visualize the variations in bacterial communities between samples using the UniFrac weighted-distance metric. The community structures of bacteria were analyzed at the genus and species levels using the Silva database (version 138) with a confidence threshold of 70%.

To identify the diurnal indicator bacteria, linear discriminant analysis effect size (LefSe) was performed with a linear discriminant analysis (LDA) score threshold of >3.0. Stream graphs are used to show the bacterial community succession during fermentation (24). The correlations (|ρ| > 0.06, *P < *0.05) among bacterial OTUs were analyzed by Spearman’s rank correlations. The OTUs with relative abundances of >0.01% were retained to construct cooccurrence networks. To compare the network structures, individual cooccurrence networks (for AM, M, and PM silages) were created using Gephi (version 0.9.2). According to the graph theory of cooccurrence networks, the average path length, average clustering coefficient, modularity, average degree, and graph density were calculated using the “*igraph*” package of R (51). Functional profiles of bacterial communities were predicted based on the 16S rRNA gene sequencing data suing Tax4Fun2 (52). Spearman’s correlation heatmaps were created using R software (version 138) to show the relationships of bacterial communities with fermentation characteristics and carbohydrate metabolism pathways.

### Statistical analysis.

Data on silage fermentation characteristics and microbial counts were analyzed using Statistical Package for Social Science 22.0 (SPSS, Inc., Chicago, IL, USA) according to a 3-by- 6 factorial treatment design (three inoculums and six fermentation times), as follows: *Y_ij_* = μ + *I_i_* + *T_j_* + (*I × T*)*_ij_* + *e_ij_*, where *Y_ij_* represents the response variable, μ is the overall mean, *I_i_* is the effect of inoculation treatments, *T_j_* is the effect of fermentation time, (*I* × *T*)*_ij_* is the effect of interaction between inoculation and fermentation time, and *e_ij_* is the random residual error. Tukey’s multiple comparison was used for the means separation. Significant differences were declared when the *P* value was <0.05.

### Data availability.

Raw sequence data have been deposited in the sequence read archive at the NCBI (https://www.ncbi.nlm.nih.gov/) under accession number PRJNA893881.
